# MicroRNA-21 and microRNA-148a affects PTEN, NO and ROS in canine leishmaniasis

**DOI:** 10.3389/fgene.2023.1106496

**Published:** 2023-04-13

**Authors:** Jéssica Henrique De Freitas, Jaqueline Poleto Bragato, Gabriela Torres Rebech, Sidnei Ferro Costa, Marilene Oliveira Dos Santos, Matheus Fujimura Soares, Flávia de Rezende Eugênio, Paulo Sérgio Patto Dos Santos, Valéria Marçal Felix De Lima

**Affiliations:** Department of Animal Clinic, School of Veterinary Medicine, Surgery and Reproduction, São Paulo State University (UNESP), Araçatuba, Brazil

**Keywords:** microRNA, ROS, NO, pten, nitrite, *Leishmania infantum*, splenic leukocytes

## Abstract

Canine Visceral leishmaniasis (CanL) poses a severe public health threat in several countries. Disease progression depends on the degree of immune response suppression. MicroRNAs (miRs) modulate mRNA translation into proteins and regulate various cellular functions and pathways associated with immune responses. MiR-21 and miR-148a can alter the parasite load and M1 macrophages are the principal cells in dogs’ leishmanicidal activity. A previous study found increased miR-21 and miR-148a in splenic leukocytes (SL) of dogs with CanL using microarray analysis and *in silico* analysis identified PTEN pathway targets. PTEN is involved in the immune regulation of macrophages. We measured PTEN and the production of reactive oxygen species (ROS) and nitric oxide (NO) before and after transfection SLs of dogs with CanL with mimic and inhibition of miR-21 and miR-148a. PTEN levels increased, NO and ROS decreased in SLs from dogs with CanL. Inhibition of miRNA-21 resulted in PTEN increase; in contrast, PTEN decreased after miR-148a inhibition. Nitrite (NO_2_) levels increased after transfection with miR-21 inhibitor but were decreased with miR-148a inhibitor. The increase in miR-21 promoted a reduction in ROS and NO levels, but miR-148a inhibition increased NO and reduced ROS. These findings suggest that miR-21 and miR-148a can participate in immune response in CanL, affecting PTEN, NO, and ROS levels.

## 1 Introduction

Visceral leishmaniasis occurs in several countries, and approximately 300,000 new cases are registered yearly. The countries with the highest recorded cases are Bangladesh, Brazil, Sudan, India, Ethiopia, and South Sudan (WHO, 2015). In Brazil, there are records of several species of *Leishmania* causing *VL*, including *L. infantum* and *L. amazonensis* in dogs (Tolezano et al., 2007; Sanches et al., 2016) and humans (Grisard et al., 2000; Araújo et al., 2020).

Dogs are reservoirs of *L. infantum* and can develop skin lesions, splenomegaly, weight loss, onychogryphosis, diarrhea, and lymphadenopathy (Tonin et al., 2016). In *Leishmania* infection, the parasite proliferates in tissues such as the liver, spleen, and bone marrow lead to functional impairment of these tissues. The spleen is where *L. infantum* can be preserved. During infection, the parasite modifies the structure of the spleen and compromises its function to favor its survival, leading to immunomodulation and disease progression (Modabberi et al., 2021).

In the spleen, granulomas can form with macrophage infiltration and rare plasma cells and lymphocytes (Moreira et al., 2016). In dogs with visceral leishmaniasis, the number of macrophages in the spleen is significantly elevated (Taffuri et al., 1996; Martini et al., 2018).

Immunosuppression favors parasite proliferation in several ways. The increased expression of the Th1 response with increased cytokines such as Interferon-γ (IFN-γ), Interleukin-2 (IL-2), and Tumor necrosis factor-α (TNF-α) might help to control the proliferation (Darrah et al., 2007). However, if the immune response is also directed toward the M2 macrophage profiles, there may be an anti-inflammatory response with increased Interleukin-10 (IL-10) (Azevedo et al., 2021), which is not beneficial for humans and mice, leading to an increased susceptibility to disease (Buxbaum, 2013). The parasite can modulate the immune response through the action of miRNAs (miRs) and direct the immune profile to a Th2 response (Bragato et al., 2022).

MicroRNAs (MiRNAs) participate in several pathophysiologies and pathways in the immune system, they are essential for developing drugs that target mRNA (Wahid et al., 2014) and can decrease or increase expression of its target gene in transcriptional or post-transcriptional level (Zhang et a., 2014; Sadakierska-Chudy, 2020). A study with miR-10a reported that miR regulates ribosome biogenesis binding to 5′Untranslated Region (UTR) and increases Ribosomal Protein (RP) production in mice (Ørom et al., 2008). Epigenetic factors or pathologies can deregulate miRNA expressions and contribute to disease development (Sadakierska-Chudy, 2020).

In Canine Visceral leishmaniasis (CanL), the parasite regulates miRNA expression in splenic leukocytes (SLs) and increases miR-21 and miR-148a expression. *In silico* analysis showed that miR-21 and miR-148a affect several pathways, including PTEN (a tumor suppressor protein) and STAT3 (a signal transducer and transcription activator). The primary direct target genes are PTEN, SOS1, and FASLG, among others (Melo et al., 2019).

MiR-21 can regulate PTEN in several diseases and it was studied in colorectal cancer (Wu et al., 2017), diabetic retinopathy (Lu et al., 2020), and endometrial carcinoma (Zhao et al., 2017). PTEN participate in proliferation, differentiation or cell growth in different cell types (Lyu et al., 2015; Kirstein et al., 2021; Wu et al., 2021), and its expression can be modulated by miRs *via* interactions with PTEN mRNA in the 3′ UTR region (Liu et al., 2019).

MiR-148a also regulates PTEN expression in macrophages infected by *Schistosoma japonicum* in mice (Giri and Cheng, 2019). In addition, overexpression of miR-148a increases reactive oxygen species (ROS) levels in macrophages through the PTEN/AKT pathway in mice (Huang et al., 2017). However, no studies demonstrate this mechanism in CanL.

MiR-21 is involved in *L. infantum* escape mechanisms by inhibiting the Th1 response in dogs with CanL (Melo et al., 2019). MiR-21 expression in SLs from dogs with CanL also regulates CD69 expression on B lymphocytes and IL-10 levels (Bragato et al., 2022). These findings suggest that *L. infantum* modulates the immune response *via* miR-21 expression as a survival mechanism (Bragato et al., 2022). In addition, the decreased parasitic load in SL following the use of the miR-148a inhibitor suggests a critical role for this miR in developing microbicidal activity in CanL, and miR148a regulates immune response as demonstrated by reducing inducible nitric oxide synthase (iNOS), TNF-α, IL-6, and IL-12 (Rebech et al., 2023).

The PTEN pathway is the target of miR-21 and miR-148a (Melo et al., 2019). PTEN controls the differentiation and activation of several immune cells and acts downstream from T- and B-cell receptors, costimulatory molecules, cytokine receptors, integrins, and growth factor receptors (Taylor et al., 2019). PTEN expression is present in M1 macrophages that promote increased inflammatory immune responses (Liu et al., 2020). Loss of PTEN activity in humans and mice is associated with cellular and humoral immune dysfunction, lymphoid hyperplasia, and autoimmunity (Taylor et al., 2019). Decreased PTEN levels and PTEN/phosphoinositide 3-kinase (PI3K)-related genetic defects are involved in the emergence of autoimmune diseases and inflammatory disorders associated with lymphoid and myeloid cells. PTEN is a PI3K antagonist that converts PI-(3,4,5) triphosphate (PIP3) to PI(4,5) (PIP2) and can control PI3P activity. PI3P is a member of the PI3K family, and its increase can lead to immunodeficiencies (Taylor et al., 2019).

The oxidative stress can play an important role in the catalytic activity of PTEN. PTEN activity is low during ROS production and it can lead to activation of the PI3K/Akt pathway (Zhang et al., 2020) and the Nrf2 transcription factor (Rojo et al., 2014) that regulates antioxidant defense, including SOD and GPx (Ma, 2013). Furthermore, high levels of ROS decreases the export of PTEN from the nucleus and lead to the accumulation of PTEN as a form of protection for the cellular DNA structure (Chang et al., 2008). iNOS expression is also involved with PTEN reduction in patients with melanoma. Nitric oxide produced from iNOS can activate the PI3K/Akt pathway and affect the catalytic activity of PTEN. The consequence of PTEN loss is a poor clinical condition in human patients affected with this tumor (Ding et al., 2021). However, increased levels of ROS and NO are important to eliminate *Leishmania* playing a protective role (Raja et al., 2016).

Splenic aspirates from patients with active *L. donovani* infection showed less mRNA PTEN expression than those from treated patients (Sudarshan et al., 2016). PTEN is involved with the immune response in leishmaniasis. PTEN-deficient macrophages showed a reduced ability to kill *L. major* in response to IFN-γ treatment, possibly because the mutant cells exhibited decreased TNF secretion that correlated with reductions in inducible iNOS expression and production (Kuroda et al., 2008). In CanL, the M1 phenotype is associated with NO, which is involved in the long-term protection of dogs against natural *L. infantum* infection and the clinical presentation of leishmaniasis (Panaro et al., 2008). NO production depends on the precursor molecule l-arginine (Jorens et al., 1993) and is induced by iNOS (Guzik et al., 2003) to perform microbicidal activity as demonstrated in human leishmaniasis (Panaro et al., 2001), mice (Mauël et al., 1991), and dogs (Vouldoukis et al., 1996). M1 macrophages also produce ROS, which participate in the innate immune response and can be produced during the oxidative explosion from Nicotinamide adenine dinucleotide phosphate (NADPH) oxidase (Saha et al., 2019).

This study aimed to evaluate the regulation of PTEN, ROS and NO levels by miR-21 and miR-148a in SLs from dogs with CanL.

## 2 Material and methods

### 2.1 Ethics committee approval

The Ethics Committee on Experimental Animal Research (COBEA) and the Ethics Committee on Animal Use (CEUA) of UNESP–São Paulo State University Júlio de Mesquita Filho, Araçatuba Campus, School of Veterinary Medicine—FMVA—approved the study, according to process 00624–2018.

### 2.2 Animals

We selected 17 adult dogs with CanL naturally infected by *L. infantum* of both sexes and various breeds and weights. The animals were aged between 2 and 5 years. Dogs with CanL were from the Araçatuba Zoonoses Control Center in the state of São Paulo. Confirmation of seropositivity to *L. infantum* was performed using an immunochromatographic test (DPP) and an indirect enzyme-linked immunosorbent assay (ELISA) (Supplementary Table S1), according to Lima et al. (2003). All dogs had at least three clinical signs of CanL, including onychogryphosis, lymphadenopathy, cachexia, and periocular dermatitis (diffuse skin or ear tip). The dogs used were classified in the moderate stage as proposed by Solano-Gallego et al. (2009) and were classified based on complete blood counts (Supplementary Tables S2 and S3) and biochemical examinations (Supplementary Table S4).

The control group consisted of 5 healthy dogs of both sexes and various breeds and weights. The animals were aged between 2 and 5 years. All dogs in this group had negative serology results according to indirect ELISA and DPP, as proposed by Lima et al. (2003). They also had complete blood counts and serum biochemistries within normal values. The diagnosis of afflicted animals was confirmed using real-time PCR (qPCR) (Ranasinghe et al., 2008) for parasitic DNA amplification and PCR-RFLP for species typing, according to Sanches et al. (2016).

### 2.3 Blood and spleen samples

5 mL of blood were drawn by jugular vein puncture and placed in tubes with an anti-coagulant (BD Vacutainer^®^, Franklin Lakes, NJ, United States of America) for the complete blood count and DNA extraction. An additional 5 mL of blood was collected and stored in a tube without an anti-coagulant for biochemical analysis and serum for the DPP and ELISA tests.

For the collection of spleen samples from the group of dogs with CanL, barbiturate anesthesia (Thiopental, Cristália Itapira, SP, Brazil) was induced by intravenous introduction, followed by infusion of 19.1% potassium chloride, according to the rules and resolution of No. 1,374, of 2 December 2020, and chapter VIII, article 27 of the Federal Council of Veterinary Medicine (Brazil). Splenic samples were then collected. Samples from control group were removed by surgical excision (Lima et al., 2012) and processed immediately for analysis.

### 2.4 Obtaining SLs

Total splenic leukocytes (SLs) (1.6 × 10⁵) were obtained from 2 cm^3^ fragments, added to 10 mL of RPMI-1640 medium (Sigma^®^, United States), and supplemented with 10% heat-inactivated fetal bovine serum, 0.03% l-glutamine, 100 IU/mL penicillin, and 100 mg/mL streptomycin. After removing debris with a cell strainer (BD Falcon Cell strainer, San Diego, CA, United States), the cell suspensions were processed with 10 mL of red blood cell lysis buffer containing 7.46 g/L of ammonium chloride (NH_4_ClO_3_) at 4 °C for 8 min, centrifuged at 2500 rpm for 9 min, and washed three times with phosphate-buffered saline at pH 7.2. RPMI-1640 medium (Sigma^®^, United States) was then added.

### 2.5 ELISA and DPP

DPP was performed according to the manufacturer’s instructions to perform screening. The sera were analyzed using indirect ELISA from of *L. infantum* promastigotes (MHOM/BR00/MER02) as the antigen. This technique was performed as described by Lima et al. (2003).

### 2.6 DNA extraction

DNA extraction from spleen samples from dogs was performed using the commercial DNAeasy^®^ kit (Qiagen, Valencia, California, 91355, United States) according to the manufacturer’s recommendations. The extracted DNA was quantified using a 260/280 nm spectrophotometer (NanoDrop Technologies ND 1000 UV/VIS, United States). The degree of purity was evaluated, and samples were stored at –20°C.

### 2.7 Determining the species of *leishmania*

The determination of *Leishmania* species was performed using PCR-RFLP (Supplementary Figure S1) to confirm *L. infantum* infection as described by Sanches et al. (2016), *L. infantum* (IOC/L0575-MHOM/BR/2002/LPC- RPV). The molecular weight marker was 100 bp (Invitrogen, Carlsbad, California 92008, United States).

### 2.8 Transfection of miR-21 and miR-148a

MiR-21 and miR-148a expression was evaluated previously in SL, and there was an increase in the expression of miR-21 (Bragato et al., 2022) and miR-148a (Rebech et al., 2023). SLs were cultured at 1.6 × 10^5^ in 24-well plates for 48 h at 37 °C in 5% CO_2_. All-Stars Negative control siRNA (Scrambled) (SCR), miR-21 mimic (5 nM), miR-148a mimic (5 nM), miR-21 inhibitor (50 nM), miR-148a inhibitor (50 nM) (miScript miRNA mimic and inhibitor Qiagen, United States) were used. Transfected cells were cultured using 3 μL of Hiperfect (Qiagen, United States) in each well, following the manufacturer’s instructions. Transfection rates were evaluated using a final concentration with 50 nM of AllStars Hs Cell Death Control siRNA reagent (Qiagen, United States), a blend of highly potent siRNAs that target genes essential for cell survival. Knocking down these genes induces a high degree of cell death. Transfection rates were measured using flow cytometry and the 7-AAD Viability Staining Solution reagent (BioLegend, United States), according to the manufacturer’s instructions. The average transfection rate for both groups was 20% (Supplementary Table S5).

The transfected cells were cultured at 37°C and 0.5% CO_2_. At 48 h after transfection, the cells were stained for NO and ROS, and cell lysates were used for PTEN evaluation. Culture supernatants from SLs were collected, centrifuged at 2500 rpm, and stored at –80°C for nitrite (NO_2_) measurement.

### 2.9 Detection of NO and ROS in SLs from infected and control dogs

To quantify NO, cell suspensions were treated with DAF2DA (2 μM) (Sigma-Aldrich, D225, São Paulo, Brazil) and incubated for 1 h at 37°C and 5% CO_2_. After labeling, samples were stored at 4°C in the dark until analysis using a flow cytometer (BD Accuri™ C5 flow cytometer), and the data were analyzed using BD Accuri™ C6 software (version 1.0.264.21). Cells (1 × 10^5^/mL) from various experimental groups were evaluated by fluorescence at 10,000 events. Cells without labeling were used as a negative control to delimit the negative populations. After excluding the debris, the cellular fluorescence of the triazole product (DAF-2T) was collected in the FL1 channel, and myeloid cells were gated separately. The mean values of FL1 were used for NO quantification.

To measure ROS levels, 10 µM H2DCFDA (Introgen-Leiden Molecular Probes) was added to cell suspensions and incubated for 1 h at 37°C and 5% CO_2_. Phorbol 12-myristate 13-acetate (PMA, 10 µM) was added 30 min before analysis for the positive ROS control. After labeling, the ROS analysis procedure was identical for NO.

### 2.10 NO_2_ determination

The Griess method determined NO_2_ concentrations in supernatant samples collected after 48 h of culture. For this procedure, we used 50 µL of the culture supernatants and 50 µL of Griess reagent (including 0.1% of NEED and sulfanilamide in 5% phosphoric acid) and maintained the mixture at room temperature for 5 min. Optical density was measured at 540 nm in 96-well plates (Spectra Count, Packard BioScience Company, Meriden, CT, United States of America). The results were compared with a standard NO_2_ concentration curve (0.3–200 µM).

### 2.11 Protein concentrations and PTEN analysis

Cell lysates were used to measure PTEN after the equalization of protein levels in samples. Protein concentrations were measured using the Pierce^®^ BCA Protein Assay Kit (Thermo Fisher Scientific). PTEN analysis was performed using the Pathscan^®^ Total PTEN Sandwich ELISA Kit (Cell Signaling Technology). PTEN is highly conserved in humans and dogs, showing 93.5% homology with *Canis lupus* familiaris (BLAST/XP_546673.2). We performed according to the manufacturer’s instructions. The optical density at 450 nm was obtained in a 96-well plate reader (Spectra Count, Packard BioScience Company, Meriden, CT, United States).

### 2.12 Statistical analysis

The data were classified as non-parametric using the Shapiro-Wilk normality test. ROS, NO, and PTEN levels were compared between the CanL and control groups using the Mann-Whitney test. PTEN, NO, ROS, and NO_2_ with mimic, inhibitor, and SCR treatments were compared using analysis of variance, the Friedman test, and Dunn’s multiple comparison test. The data were classified as parametric using the Shapiro-Wilk. Mean ROS levels from non-transfected cells were compared with stimulated PMA cells using the *t*-test. NO_2_ and NO levels of miR-148a mimic, inhibitor, and SCR treatments in CanL were compared using analysis of variance with Dunnett´s multiple comparison test. Differences were considered significant when *p* < 0.05. Statistical analyses and graphs were generated using Graph Pad Prism 6 (Graph Pad Software, Inc. CA. United States).

## 3 Results

### 3.1 NO, ROS and PTEN concentrations in SLs of dogs with CanL

**FIGURE 1:**
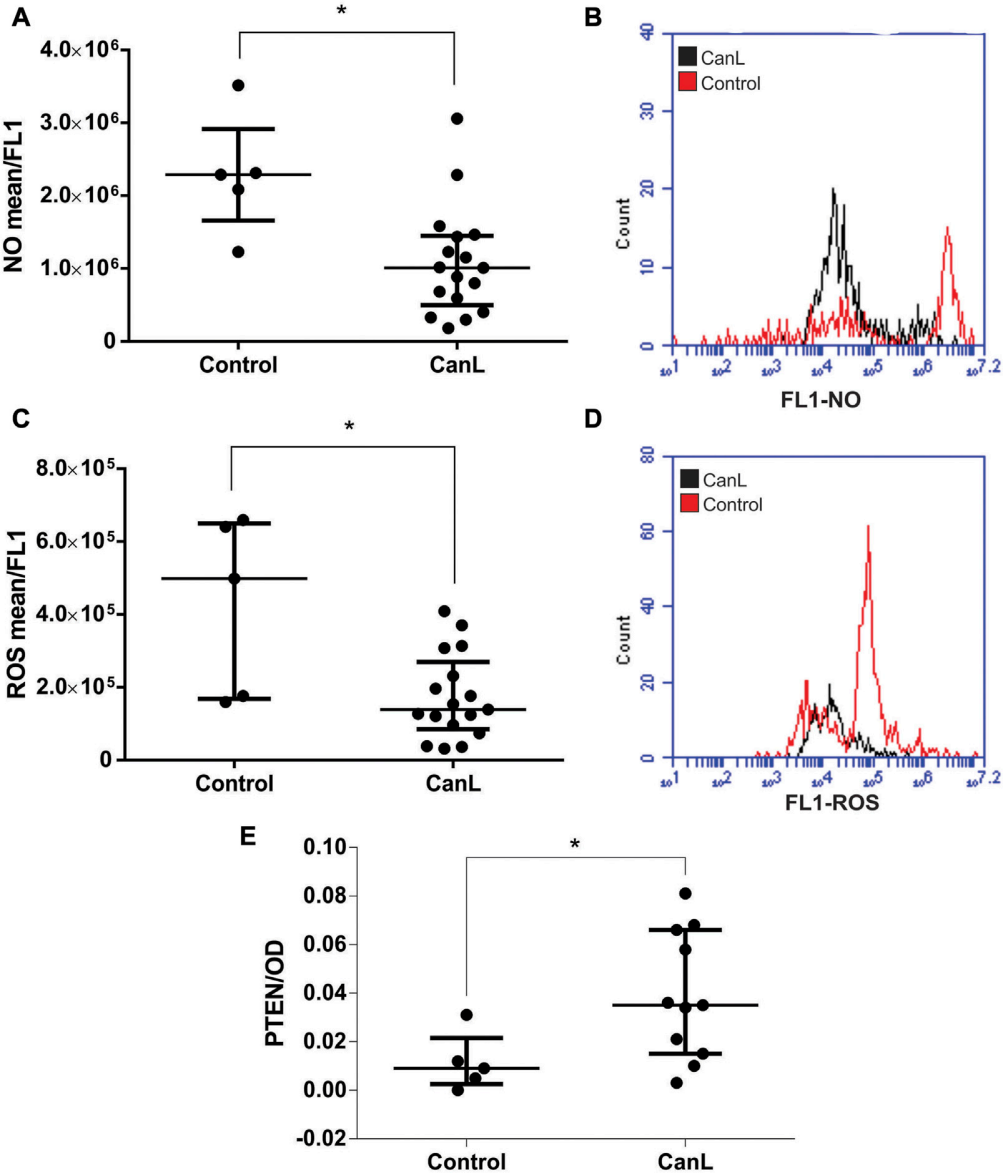
NO **(A)** and ROS **(C)** in SLs from control group and dogs with CanL. PTEN **(E)** in total cell lysate samples in control group and dogs with CanL by sandwich ELISA from Pathscan^®^ kit. Data are expressed as median and interquartile range. Mann-Whitney test (^*^ *p* < 0.05). A representative overlay histogram shows an example of NO **(B)** and ROS **(D)** production in SLs from control group and dogs with CanL. The black represents dogs with CanL and the red represents control group.

NO production was lower in SLs from dogs with CanL than in the control group (Figure 1A) (*p* = 0.0063). ROS production was lower in SLs from dogs with CanL than in the control (Figure 1C) (*p* = 0.0246), and PMA (positive control) increased ROS production in the CanL group (Supplementary Table S6). We measured PTEN from whole cell lysates, and levels were higher in SLs from dogs with CanL than from control (Figure 1E) (*p* = 0.0270). The dogs from the control group were tested serologically negative for *L. infantum*. A representative histogram of basal NO and ROS production in SLs from dogs with CanL and a control are shown in Figures 1B–D, respectively. NO and ROS production can stablish leishmanicidal activity (Novais et al., 2014; Olekhnovitch and Bousso, 2015), suggesting that activity is impaired in spleen from dogs with CanL.

### 3.2 Effect of miR-21 and miR-148a on PTEN concentration in SLs of dogs with CanL

**FIGURE 2:**
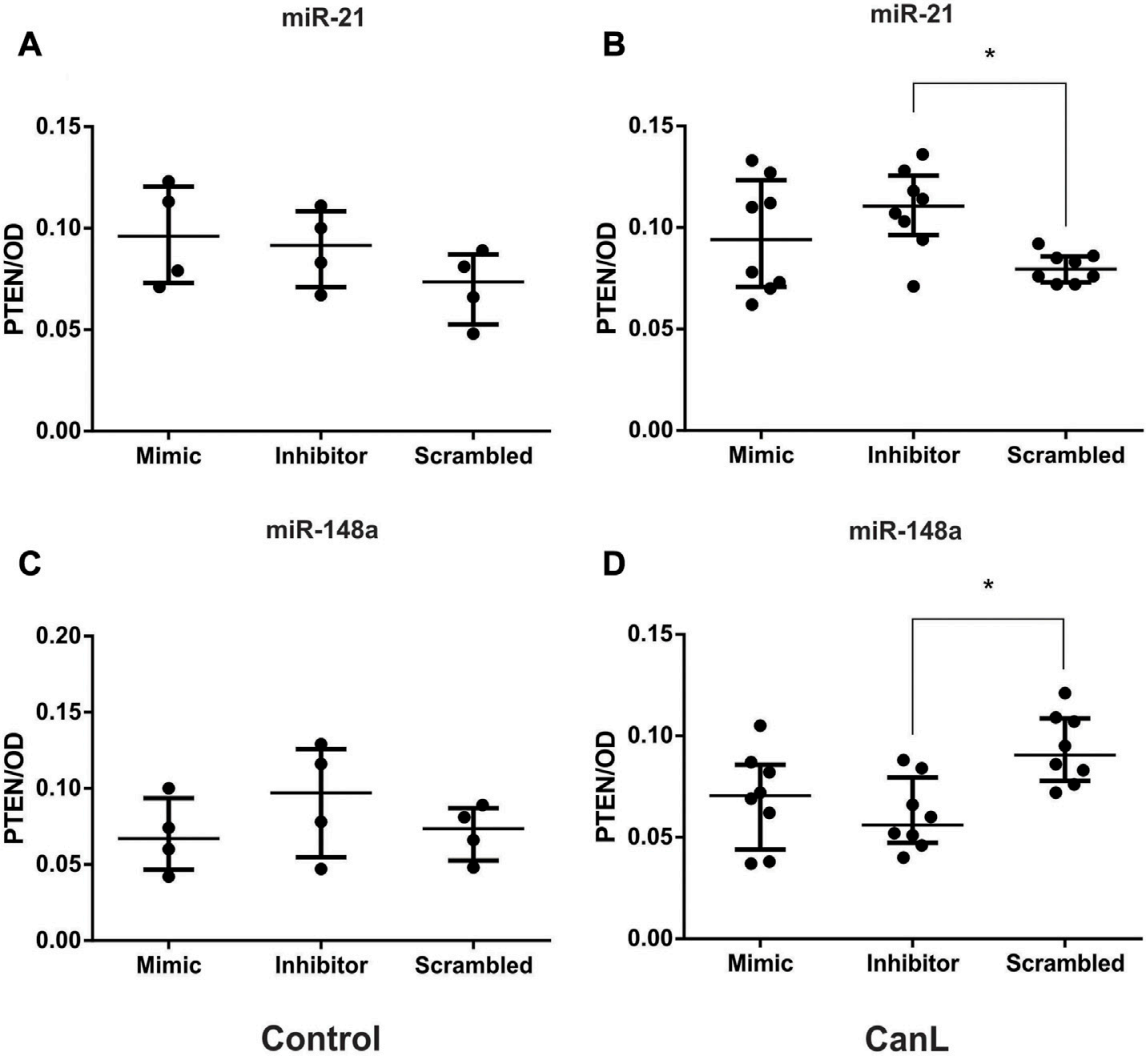
PTEN in cell lysates after transfection with miR-21 mimic and inhibitor in control dogs **(A)** and dogs with CanL **(B)**; miR-148a mimic and inhibitor in control dogs **(C)** and dogs with CanL **(D)**. Data are expressed as median and interquartile range. Analysis of variance with multiple comparisons (^*^ *p* < 0.05).

After SL transfection with miR-21 mimic and inhibitor, and miR-148a mimic and inhibitor, no significant difference was observed in PTEN level in the control group, when compared with SCR treated cells (Figures 2A–C) (*p* > 0.05). The transfection with miR-21 inhibitor, there was more increased PTEN concentration in cell lysates of dogs with CanL then when SCR oligonucleotides used as a transfection control (Figure 2B) (*p* = 0.0302), in contrast, in SL transfected with miR-148a inhibitor, there was less PTEN than in samples from dogs with CanL, than when SCR oligonucleotides used as a transfection control (Figure 2D) (*p* = 0.070). Based on sequence homology with humans, we can infer that miR-148a binds the 3′UTR region of the PTEN transcript based on TargetScan analysis. We also performed mirDIP analysis, demonstrating target scores of miR-21 and miR-148a (Supplementary Table S7).

### 3.3 Effect of miR-21 and miR-148a on NO concentration in SLs of dogs with CanL

**FIGURE 3:**
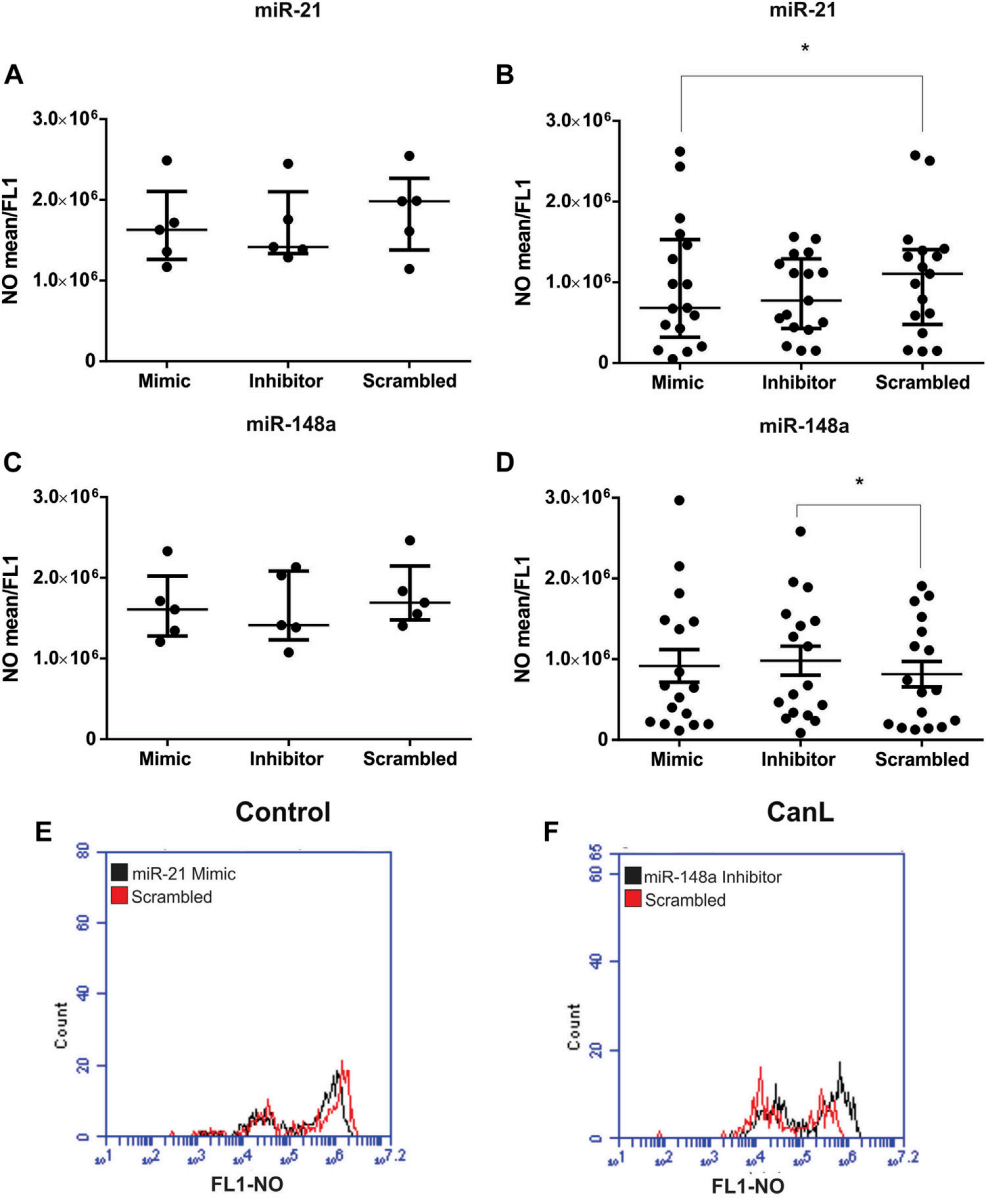
NO in SLs after transfection with miR-21 mimic and inhibitor in control dogs **(A)** and dogs with CanL **(B)**, and miR-148a mimic and inhibitor in control dogs **(C)** and dogs with CanL **(D)**. Data are expressed as the median and interquartile range in **(A)**, **(B)** and **(C)**. Data are expressed as the mean and standard error in **(D)**. The representative overlay histogram of the action of miR-21 mimic in inhibiting NO production **(E)** and the increased NO production by the inhibitor of miR-148a **(F)** in SLs of dogs with CanL. The treatment with mimic and inhibitor is in black and the SCR is in red. Friedman test and analysis of variance with multiple comparisons (*p < 0.05).

In this study, we observed that NO production was lower in SLs from dogs with CanL that in the control. Next, we evaluated the effect of miR-21 and miR-148a transfection of SLs with mimic and inhibitor from dogs with the control group and CanL on the regulation of NO production. Transfected SLs were cultured at 37 °C and 5% CO_2_ for 48 h, and NO production was evaluated using flow cytometry. We did not find significant differences in the control group following treatment with the miR-21 mimic and inhibitor and miR-148a mimic and inhibitor, when compared with SCR treated cells (Figures 3A–C) (*p* > 0.05). By contrast, SLs from dogs with CanL transfected with miR-21 mimic, there was less NO than when SCR oligonucleotides were used as a transfection control (*p* = 0.0469) (Figure 3B). However, in SLs transfected with the miR-148a inhibitor, there was more NO production in dogs with CanL than when SCR oligonucleotides were used as a transfection control (Figure 3D) (*p* = 0.0462). A representative overlay histogram displays the action of mimic inhibiting NO production by miR-21 (Figure 3E) and the increase of NO production by miR-148a inhibitor (Figure 3F) in SLs from dogs with CanL.

### 3.4 Effect of miR-21 and miR-148a on NO_2_ production in SLs of dogs with CanL

**FIGURE 4:**
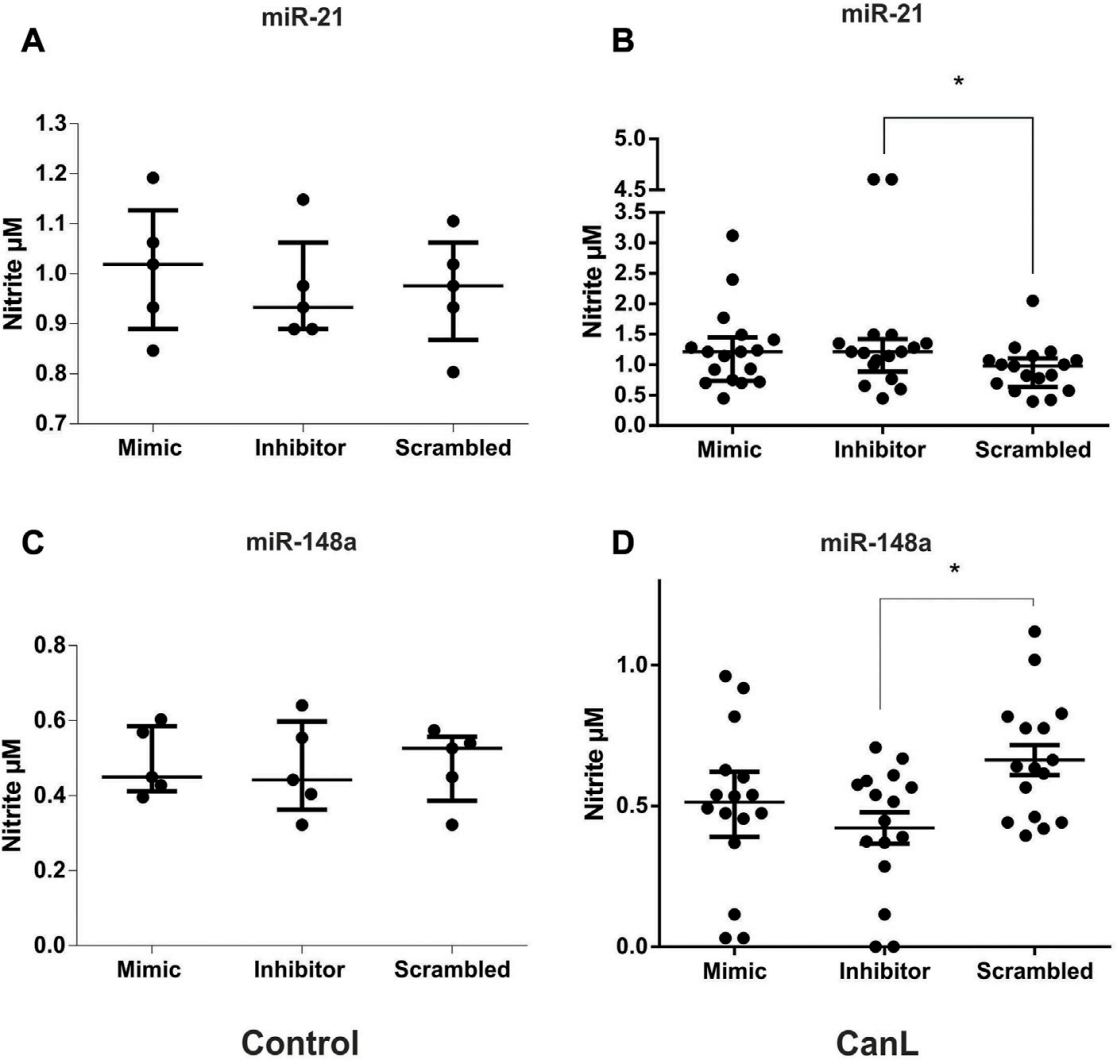
NO_2_ in SLs after transfection with miR-21 mimic and inhibitor in control dogs **(A)** and dogs with CanL **(B)**, and miR-148a mimic and inhibitor in control dogs **(C)** and dogs with CanL **(D)**. Data are expressed as the median and interquartile range in **(A)**, **(B)** and **(C)**. Data are expressed as the mean and standard error of the mean in **(D)**. We used the Friedman test in A, and analysis of variance (with multiple comparisons) and Dunnett’s multiple comparison test in B (*p < 0.05).

We evaluated the effect of transfection with miR-21 mimic and inhibitor and miR-148a mimic and inhibitor on NO_2_ production in SLs from dogs of the control group and CanL. Transfected SLs were cultured at 37 °C and 5% CO_2_ for 48 h, and production was measured using the Greiss method in culture supernatants. No significant difference was observed in the control group following treatment with the miR-21 mimic and inhibitor and miR-148a mimic and inhibitor in NO_2_ production, when compared with SCR treated cells (Figures 4A–C) (*p* > 0.05). After transfection with miR-21 inhibitor from SLs of dogs with CanL, there was a more significant NO_2_ concentration in culture supernatants, than with SCR oligonucleotides used as a transfection control (Figure 4B) (*p* = 0.0359). However, the transfection with miR-148a inhibitor in SL, there was less NO_2_ in culture supernatants in dogs with CanL than when SCR (Figure 4D) (*p* = 0.0069).

### 3.5 ROS concentration and the effect of miR-21 and miR-148a on the regulation of its production in SLs of dogs with CanL

**FIGURE 5:**
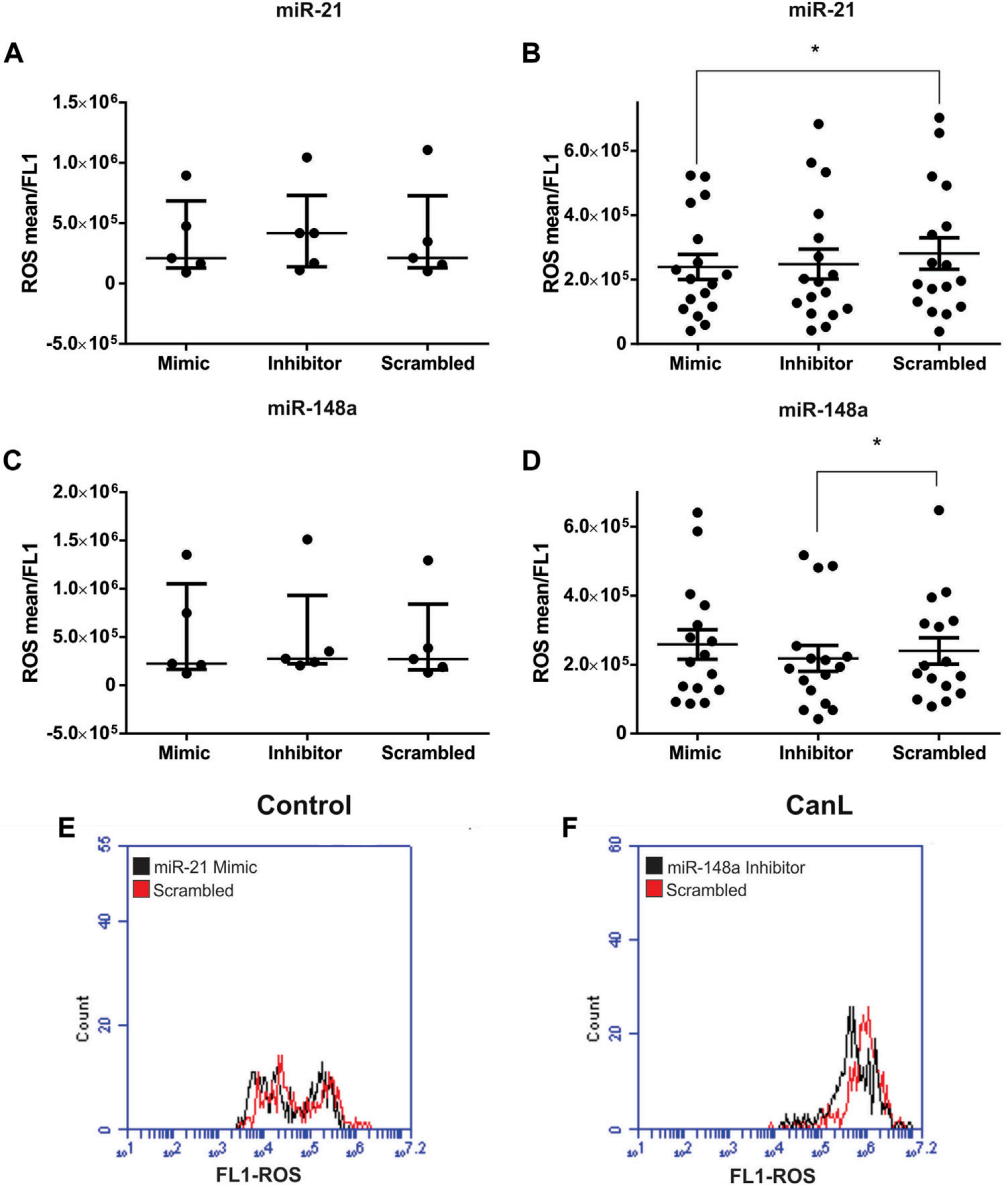
ROS in SLs after transfection with miR-21 mimic and inhibitor in control dogs **(A)** and dogs with CanL **(B)**, and miR-148a mimic and inhibitor in control dogs **(C)** and dogs with CanL **(D)**. Data are expressed as the median and interquartile range in **(A)**, **(B)** and **(C)**, and as the mean and standard error in **(D)**. Representative histogram of the action of mimic on ROS production by miR-21 **(E)** and ROS inhibition by miR-148a inhibitor **(F)** in SLs from dogs with CanL. The treatment with mimic and inhibitor is represented by black and the SCR by red. We performed the Friedman test and analysis of variance with multiple comparisons (^*^ *p* < 0.05).

In this study, we observed that ROS production was lower in SLs from dogs with CanL than in the control. Next, we evaluated the effect of miR-21 and miR-148a transfection on ROS production in SL from dogs with the control group and CanL with mimic and inhibitor. No difference was observed after transfection with miR-21 mimic and inhibitor, and miR-148a mimic and inhibitor in the control group, when compared with SCR treated cells (Figures 5A–C) *(p* > 0.005). However, the transfection with miR-21 mimic gave rise to a decrease in ROS production in dogs with CanL compared to SCR oligonucleotides used as a transfection control (Figure 5B) (*p = 0.0469). After transfection with miR-148a inhibitor in SLs, ROS production was reduced in dogs with CanL, when compared to SCR (Figure 5D) (*p* = 0.0468). A representative overlay histogram of the action of mimic on the inhibition of ROS production by miR-21 (Figure 5E) and the inhibition of ROS production by the miR-148a inhibitor (Figure 5F) was shown in SLs from dogs with CanL.

## 4 Discussion

SLs from dogs with CanL show increased miR-21 and miR-148 targeting PTEN (Melo et al., 2019). PTEN has several regulatory roles in the immune system, including shifting the macrophage profile from M2 to M1 (Sahin et al., 2014; Liu et al., 2020). To clarify the regulatory role of miR-21 and miR-148a targeting PTEN in CanL, SLs from dogs with CanL were transfected with mimic and inhibitor of miR-21 and miR-148a, and we measured PTEN, NO, and ROS levels.

PTEN concentrations increased in lysates from SLs from dogs with CanL. In contrast, less PTEN mRNA was observed during the active stage of *L. donovani* infection in human splenic cells (Sudarshan et al., 2016). This difference can be related to mRNA detection instead of protein, as in our study.

High PTEN levels were associated with decreased NO and ROS production by SLs, suggesting a possible inhibition of inflammatory response in CanL. Higher numbers of M2 macrophages were observed in spleens in CanL (Moreira et al., 2016). M2 polarization of macrophages with decreased NO and ROS possibly favors parasite survival. These findings suggest that *L. infantum* modulates PTEN, NO, and ROS levels. Nevertheless, more studies are needed to clarify this mechanism.

In our study, NO concentration was lower in SLs in the CanL group, confirming a result previously observed in CanL (Martini et al., 2018). NO production was associated with microbicidal activity in CanL (Vouldoukis et al., 1996), and its low production by macrophages could explain the high parasite replication observed in the spleens of the CanL group.

We observed low concentrations of ROS in SLs in CanL. Similarly, splenic macrophages from mice infected with *L. donovani* showed low production of ROS (Ball et al., 2011). In peripheral blood mononuclear cells from humans with visceral leishmaniasis, the parasite decreased the expression of NADPH oxidase, preventing respiratory bursts (Kumar et al., 2002). The parasite expresses lipophosphoglycan, glycoinositol phospholipids, and ceramides that inhibit the Protein Kinase C activation pathway associated with ROS production (Atayde et al., 2016). In CanL, this low production of ROS might facilitate disease progression. Our findings suggest that *L. infantum* regulates host molecules to decrease microbicidal activity.

Low ROS and NO in SLs from dogs with CanL were associated with high PTEN levels in cell lysates. Upregulation of PTEN causes modulation of PI3K/AKT (Protein Kinase B) signaling to reduce ROS generation (Xu et al., 2011). The PI3K/AKT/PTEN pathway is involved in the defense mechanisms against *L. amazonensis* (Calegari-Silva et al., 2015), and PTEN negatively regulates the activity of PI3K/AKT signaling by converting PIP3 to PIP2, by which PTEN exerts its tumor-suppressive effect. Notably, the intracellular growth of the parasite was impaired during PI3K/Akt signaling inhibition and in macrophages with knocked-down Akt 1 expression. iNOS expression were decreased when PI3K/Akt pathway were high expressed in macrophages infected with *L. amazonensis* (Calegari-Silva et al., 2015). In addition, PTEN is also inactivated during the oxidative stress process by ROS (Leslie et al., 2003) and NO production in humans (Kwak et al., 2010). Low PTEN can lead to the activation of Nrf2 (Taguchi et al., 2014) that is responsible to modulate the antioxidative pathway and favors infection by *L. infantum* (Vivarini and Lopes, 2020). Our findings suggest that *L. infantum* regulates PTEN in CanL. However, it is necessary to investigate the participation of the PI3K/AKT pathway in dogs with CanL.

PTEN negatively regulated the expression of pro-inflammatory cytokines such as TNF-α in an experimental model of adjuvant-induced arthritis (Li et al., 2019). PTEN was associated with the downregulation of inflammatory cytokines in humans (Yan et al., 2019). In dogs with CanL, TNF is markedly reduced with increased parasite load (Cavalcanti et al., 2015). TNF-α enhances NO production in canine macrophages (Pinelli et al., 2000). Therefore, probable PTEN may regulate cytokines; however, this finding must be confirmed in CanL.

Inhibition of miR-21 in SLs increased PTEN concentrations in cell lysate from SLs in CanL. When bone marrow-derived stem cells from dogs were transfected with the miRNA-21 inhibitor, PTEN was upregulated (Yang et al., 2019). Studies with human cancer showed that downregulation of miR-21 increased PTEN expression, inhibited PI3K/AKT activity, promoted apoptosis, and reduced proliferation (Meng et al., 2007; Liu et al., 2019). In human cells, miR-21 modulated gene expression directly at the PTEN 3′-UTR (Meng et al., 2007), this mechanism could occur in dogs with CanL. Molecular studies are necessary to test this hypothesis. We suggest that *L. infantum* has the potential to change immune response through miR-21, which has the PTEN pathway as a target.

We showed an increased PTEN by miR-21 inhibition in SLs occurred while nitrites increased. The miR-21 mimic reduced NO in our study, *Leishmania* parasites are killed by macrophages activated by NO-dependent mechanism (Vouldoukis et al., 1996). Interestingly *in vitro* study shows PTEN-deficient macrophages reduce their ability to kill *L. major* in response to IFN-γ treatment, possibly because the mutant cells exhibited decreased TNF secretion that correlated with reductions in inducible iNOS expression and NO production (Kuroda et al., 2008). In our study, high PTEN associated with inhibited miR-21 induced high NO_2_, suggesting a role for PTEN and miR-21 in parasitic control in CanL.

We observed low ROS concentration in SLs in CanL after transfection with miR-21 mimic. In a *L. donovani* infection mice model, the spleens of miR-21 knockout mice expressed higher transcripts of M2 macrophages (Varikuti et al., 2021), which are responsible for producing low ROS, impairing a defense mechanism that controls the disease in mice (Gonçalves et al., 2018). Our findings suggest that ROS is regulated by miR-21. However, further studies are needed to clarify these mechanisms and understand how this phenomenon affects immune response in CanL.

In the present study, inhibition of miR-148a in SLs reduce PTEN. Inhibition of miR-21 increased PTEN concentration in cell lysates from SLs in CanL. The luciferase assay showed that PTEN is a target of miR-148a in mice, and bioinformatics analyses showed that miR-148a binds the 3’ UTR of the PTEN gene (Qingjuan et al., 2016). We performed bioinformatics analysis and observed that miR-21 could regulate the PTEN pathway, while miR-148a has PTEN as a direct target. These findings suggest that PTEN expression can be regulated by more than one miRNA in CanL.

We observed SLs transfected with the miR-148a inhibitor there was more significant NO production, in contrast miR-148a mimic also decrease iNOS in SL in CanL (Rebech et al., 2023), and similar study observed that overexpression of miR-148a in peripheral blood cells of rats was associated with low iNOS (Yin et al., 2021). NO production by macrophages participates in the elimination of *L. infantum* and can be involved in Th1-type immune responses (Reza et al., 2019). These findings suggest that miR-148a participates in NO regulation and that *L. infantum* could use miR-148a expression to favor its survival.

The miR-148a inhibitor decreased ROS concentrations in dogs with CanL. Interestingly, in mice bone marrow, the overexpression of miR-148a-3p in macrophages promoted excessive production of ROS and was responsible for the bactericidal activity of M1 macrophages through the PTEN/AKT pathway (Huang et al., 2017). ROS production from macrophages during *L. donovani* infection is responsible for eliminating the parasite (Das et al., 2021). The PI 3-Kinase pathway promotes oxidative stress with ROS production and can involve PTEN (Leslie et al., 2003). *L. infantum* could use this pathway to decrease ROS levels in CanL as a survival mechanism. We suggest further studies to confirm this finding.

We conclude that miR-21 and miR-148a participate in the immune responses against *L. infantum* by targeting the PTEN pathway. The parasite *L. infantum* can regulate ROS and NO production through miR-21 and miR148a expression for its survival in the host. These findings may contribute to future therapies targeting these miRs for treating CanL.

## Data Availability

The original contributions presented in the study are included in the article/Supplementary Material, further inquiries can be directed to the corresponding author.
