# Supplementary material for: MicroRNA-21 and microRNA-148a affects PTEN, NO and ROS in canine leishmaniasis
Source: Front Genet. 2023 Apr 13;14:1106496. doi: 10.3389/fgene.2023.1106496 (PMC10137164; doi:10.3389/fgene.2023.1106496)
Supplement: Supplementary file 1 [file Table1.DOCX]

**Table 1. Serology to detect anti-*leishmania sp* antibodies and principal clinical signs of CanL and healthy dogs.**

| Animal | D.O. ELISA | Sex | Clinical signs | DPP | PCR |
| --- | --- | --- | --- | --- | --- |
| CanL 1 | 1.132 | F | Onychogryphosis, skin lesions, cachexia, seborrhea. | + | + |
| CanL 2 | 1.060 | M | Lymphadenopathy, onychogryphosis, cachexia, and skin lesions. | + | + |
| CanL 3 | 0.920 | M | Cachexia, seborrhea and skin lesions. | + | + |
| CanL 4 | 0.473 | M | Lymphadenopathy, skin lesions, hepatosplenomegaly. | + | + |
| CanL 5 | 1.373 | F | Onychogryphosis, cachexia, alopecia, skin lesions. | + | + |
| CanL 6 | 1.207 | M | Lymphadenopathy, onychogryphosis, cachexia, alopecia, paw lesions. | + | + |
| CanL 7 | 1.288 | F | Lymphadenopathy, onychogryphosis, seborrhea, alopecia, periocular lesion, hepatosplenomegaly. | + | + |
| CanL 8 | 1.267 | F | lymphadenopathy, onychogryphosis, seborrhea, alopecia, skin lesions, hepatosplenomegaly. | + | + |
| CanL 9 | 1.048 | F | Lymphadenopathy, onychogryphosis, periocular lesion, hepatosplenomegaly. | + | + |
| CanL 10 | 0.968 | F | lymphadenopathy, onychogryphosis, cachexia | + | + |
| CanL 11 | 0.649 | F | Onychogryphosis, cachexia, seborrhea, body lesions, ear, skin, snout, paws | + | + |
| CanL 12 | 1.219 | M | Lymphadenopathy, cachexia, seborrhea, lesions on the body, ear, skin, snout, paws, periocular and hepatosplenomegaly | + | + |
| CanL 13 | 1.192 | M | Lymphoadenomegaly, cachexia, seborrhea, ear, snout and periocular lesions | + | + |
| CanL 14 | 0.666 | M | Cachexia, ectoparasites, ear, skin, paws and periocular lesions | + | + |
| CanL 15 | 1.072 | F | Cachexia, skin and periocular lesions | + | + |
| CanL 16 | 1.007 | F | Onychogryphosis, seborrhea, ear and periocular lesions | + | + |
| CanL 17 | 0.759 | M | Onychogryphosis, paw lesions and seborrhea | + | + |
| 1 | 0.062 | F | - | - | - |
| 2 | 0.026 | M | - | - | - |
| 3 | 0.147 | F | - | - | - |
| 4 | 0.028 | M | - | - | - |
| 5 | 0.071 | F | - | - | - |
