# Supplementary material for: MicroRNA-21 and microRNA-148a affects PTEN, NO and ROS in canine leishmaniasis
Source: Front Genet. 2023 Apr 13;14:1106496. doi: 10.3389/fgene.2023.1106496 (PMC10137164; doi:10.3389/fgene.2023.1106496)
Supplement: Supplementary file 2 [file Table7.docx]

| MiR-21 | Target | MiRDIP (Integrated Score) |
| --- | --- | --- |
|  | BMPR2 | 0.85 |
|  | FASLG | 0.76 |
|  | PIK3R1 | 0.85 |
|  | TGFBR2 | 0.80 |
|  |  |  |
| MiR-148a | Target | MiRDIP (Integrated Score) |
|  | NRAS | 0.84 |
|  | PTEN | 0.89 |
|  | SOS2 | 0.92 |

**Table 7. MiRDIP analysis.**
