# Supplementary material for: MicroRNA-21 and microRNA-148a affects PTEN, NO and ROS in canine leishmaniasis
Source: Front Genet. 2023 Apr 13;14:1106496. doi: 10.3389/fgene.2023.1106496 (PMC10137164; doi:10.3389/fgene.2023.1106496)
Supplement: Supplementary file 3 [file Table2.DOCX]

**Table 2. Red blood count in CanL and healthy dog groups**

| **Animal** | **RBC** | **Hemoglobin** | **GV** | **MCV** | **MCHC** |
| --- | --- | --- | --- | --- | --- |
| **Reference** | **5.5-8.5 x 10^12^/L** | **12.0-18.0g/dl** | **37-55**  **%** | **60-77**  **fL** | **32-36**  **%** |
| CanL 1 | 4,25 | 10,1 | 29 | 68,24 | 34,83 |
| CanL 2 | 3,88 | 8,6 | 26 | 67,01 | 33,08 |
| CanL 3 | 4,05 | 7,8 | 25 | 61,73 | 31,2 |
| CanL 4 | 2,93 | 6,7 | 22 | 75,09 | 30,45 |
| CanL 5 | 2,95 | 6,5 | 18 | 61,02 | 36,11 |
| CanL 6 | 4,3 | 9,5 | 27 | 62,79 | 35,19 |
| CanL 7 | 3,34 | 6,2 | 18 | 53,89 | 34,44 |
| CanL 8 | 4,06 | 10 | 28 | 68,97 | 35,71 |
| CanL 9 | 2,1 | 4,1 | 12 | 57,14 | 34,17 |
| CanL 10 | 3,81 | 8,7 | 25 | 65,62 | 34,8 |
| CanL 11 | 5,32 | 11,4 | 34 | 63,91 | 33,53 |
| CanL 12 | 4,55 | 10,5 | 32 | 70,33 | 32,81 |
| CanL 13 | 4,91 | 11,6 | 34 | 69,25 | 34,12 |
| CanL 14 | 5,5 | 14 | 39 | 70,91 | 35,9 |
| CanL 15 | 4,55 | 10,4 | 32 | 70,33 | 32,5 |
| CanL 16 | 5,77 | 12,3 | 37 | 64,12 | 33,24 |
| CanL 17 | 5,33 | 11,7 | 35 | 65,67 | 33,43 |
| 1 | 7,89 | 17,8 | 53 | 67,17 | 33,58 |
| 2 | 6,78 | 16,9 | 49 | 72,27 | 34,49 |
| 3 | 7,89 | 17,8 | 52 | 65,91 | 34,23 |
| 4 | 6,78 | 17,3 | 50 | 73,75 | 34,6 |
| 5 | 7,44 | 17,1 | 51 | 68,55 | 33,53 |

CanL: Canine Leishmaniasis; RBC: Red blood cells; GV: Globular volume; MCV: Mean corpuscular volume; MCHC: Mean corpuscular hemoglobin concentration.
