# Supplementary material for: MicroRNA-21 and microRNA-148a affects PTEN, NO and ROS in canine leishmaniasis
Source: Front Genet. 2023 Apr 13;14:1106496. doi: 10.3389/fgene.2023.1106496 (PMC10137164; doi:10.3389/fgene.2023.1106496)
Supplement: Supplementary file 4 [file DataSheet1.PDF]

Supplementary Figure 1. PCR-RFLP analysis in CanL samples.

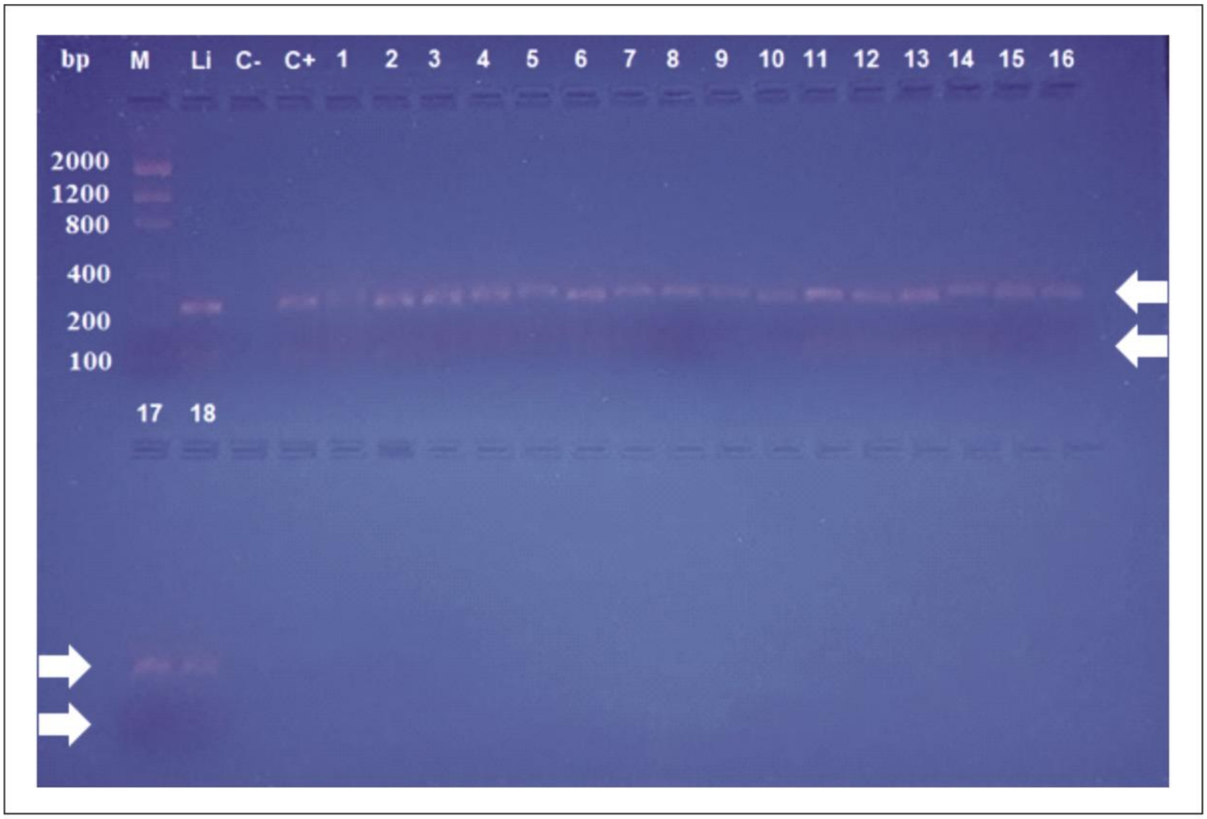

Supplementary Figure 1: Restriction fragment length polymorphism analysis with ITS1-PCR fragments amplified from DNA samples using Hae III. M: molecular marker (100 bp); C-: Negative control (water); C+: positive control; Li: *Leishmania infantum* (IOC / L0575-MHOM / BR / 2002 / LPC – RPV); The samples were identified as one to 18 and are identical to the *L. infantum* profile.
