# Supplementary material for: MicroRNA-21 and microRNA-148a affects PTEN, NO and ROS in canine leishmaniasis
Source: Front Genet. 2023 Apr 13;14:1106496. doi: 10.3389/fgene.2023.1106496 (PMC10137164; doi:10.3389/fgene.2023.1106496)
Supplement: Supplementary file 5 [file Table5.DOCX]

**Table 5. Transfection rate.**

| Animal | Transfection rate % |
| --- | --- |
| CanL 1 | 17 |
| CanL 2 | 11 |
| CanL 3 | 41 |
| CanL 4 | 30 |
| CanL 5 | 25 |
| CanL 6 | 42 |
| CanL 7 | 20 |
| CanL 8 | 22 |
| CanL 9 | 20 |
| CanL 10 | 22 |
| CanL 11 | 17 |
| CanL 12 | 26 |
| CanL 13 | 16 |
| CanL 14 | 22 |
| CanL 15 | 16 |
| CanL 16 | 16 |
| CanL 17 | 28 |
| 1 | 24 |
| 2 | 13 |
| 3 | 12 |
| 4 | 10 |
| 5 | 48 |
