# Supplementary material for: MicroRNA-21 and microRNA-148a affects PTEN, NO and ROS in canine leishmaniasis
Source: Front Genet. 2023 Apr 13;14:1106496. doi: 10.3389/fgene.2023.1106496 (PMC10137164; doi:10.3389/fgene.2023.1106496)
Supplement: Supplementary file 6 [file Table3.DOCX]

**Table 3: White blood cells and platelets count in CanL and Healthy dogs.**

| **Animals** | **Leukocytes** | **Neutrophils** | **Eosinophils** | **Basophils** | **Monocytes** | **Lymphocytes** | **Platelets** |
| --- | --- | --- | --- | --- | --- | --- | --- |
| **Reference** | **6.0-17.0 x 10^9^/L** | **3000-11500 x 10^6^/L** | **150-1250 x 10^6^/L** | **Rare** | **150-1350 x 10^6^/L** | **1000-4800 x 10^6^/L** | **160-400 x 10^3^/L** |
| CanL 1 | 5,4 | 4212 | 108 | 0 | 486 | 594 | 220 |
| CanL 2 | 13,3 | 11172 | 0 | 0 | 133 | 1995 | 160 |
| CanL 3 | 9,9 | 6831 | 198 | 0 | 396 | 2475 | 220 |
| CanL 4 | 7,1 | 4686 | 0 | 0 | 284 | 2130 | 300 |
| CanL 5 | 9,3 | 6417 | 186 | 0 | 837 | 1860 | 280 |
| CanL 6 | 7,2 | 4320 | 72 | 0 | 288 | 1800 | 140 |
| CanL 7 | 8,1 | 5022 | 0 | 0 | 81 | 2997 | 180 |
| CanL 8 | 8,5 | 6035 | 0 | 0 | 425 | 2040 | 200 |
| CanL 9 | 3 | 2100 | 90 | 0 | 30 | 780 | 220 |
| CanL 10 | 14,6 | 10950 | 146 | 0 | 146 | 3358 | 280 |
| CanL 11 | 8 | 6080 | 160 | 0 | 160 | 1600 | 400 |
| CanL 12 | 7,8 | 5226 | 234 | 0 | 78 | 2262 | 280 |
| CanL 13 | 10,8 | 8748 | 0 | 0 | 324 | 1728 | 380 |
| CanL 14 | 7,4 | 5772 | 222 | 0 | 444 | 962 | 400 |
| CanL 15 | 7,1 | 4047 | 0 | 0 | 142 | 2911 | 180 |
| CanL 16 | 17 | 12240 | 1870 | 0 | 850 | 2040 | 280 |
| CanL 17 | 6 | 4020 | 120 | 0 | 60 | 1800 | 160 |
| 1 | 16,2 | 10692 | 1250 | 0 | 1496 | 2754 | 200 |
| 2 | 15,7 | 10048 | 2041 | 0 | 628 | 2983 | 300 |
| 3 | 12,2 | 6588 | 122 | 0 | 610 | 4800 | 320 |
| 4 | 16,2 | 10854 | 1296 | 0 | 324 | 3726 | 220 |
| 5 | 10,3 | 6077 | 103 | 0 | 515 | 3605 | 220 |

CanL: Canine leishmaniasis.
