# Supplementary material for: MicroRNA-21 and microRNA-148a affects PTEN, NO and ROS in canine leishmaniasis
Source: Front Genet. 2023 Apr 13;14:1106496. doi: 10.3389/fgene.2023.1106496 (PMC10137164; doi:10.3389/fgene.2023.1106496)
Supplement: Supplementary file 7 [file Table4.DOCX]

| **Animals** | **Albumin** | **Globulin** | **Total protein** | **Creatinine** | **Urea** | **ALT** | **Alkaline Phosphatase** | **GGT** |
| --- | --- | --- | --- | --- | --- | --- | --- | --- |
| **Reference** | **2.6-3.3**  **g/L** | **2.7-4.4**  **g/L** | **5.4-7.1**  **g/L** | **0.5-1.5**  **mg/dL** | **10.03-50.03**  **mg/dL** | **21-102**  **UI/L** | **20-156**  **UI/L** | **1.2-6.4**  **UI/L** |
| CanL 1 | 1,6 | 8,8 | 10,4 | 0,6 | 42 | 26 | 66 | 5,9 |
| CanL 2 | 1 | 6,6 | 7,6 | 0,7 | 33 | 42 | 243 | 2,9 |
| CanL 3 | 1,8 | 9 | 10,8 | 0,7 | 30 | 37 | 75 | 6 |
| CanL 4 | 1,9 | 6,5 | 8,4 | 1 | 45 | 21 | 24 | 1,3 |
| CanL 5 | 0,94 | 6,1 | 7 | 0,8 | 70 | 21 | 29 | 4,9 |
| CanL 6 | 1,1 | 5,2 | 6,3 | 1 | 60 | 21 | 109 | 1,9 |
| CanL 7 | 1,5 | 5,6 | 7,1 | 0,5 | 30 | 37 | 22 | 2,9 |
| CanL 8 | 1,9 | 6,8 | 8,7 | 0,7 | 49 | 92 | 92 | 1 |
| CanL 9 | 2,2 | 4,6 | 6,8 | 0,5 | 35 | 70 | 70 | 1,5 |
| CanL 10 | 1,2 | 7,6 | 8,8 | 1,5 | 120 | 30 | 42 | 1,4 |
| CanL 11 | 2 | 8,4 | 10,4 | 0,8 | 42 | 25 | 23 | 2,8 |
| CanL 12 | 1,6 | 7,9 | 9,5 | 1 | 51 | 37 | 67 | 2,1 |
| CanL 13 | 1,8 | 7,6 | 9,4 | 0,9 | 35,4 | 15 | 57 | 6 |
| CanL 14 | 3 | 4,2 | 7,2 | 0,7 | 32 | 160 | 42 | 4 |
| CanL 15 | 1,2 | 6,1 | 7,3 | 0,8 | 61 | 94 | 268 | 9 |
| CanL 16 | 2,1 | 6,7 | 8,8 | 0,9 | 23 | 18 | 97 | 4 |
| CanL 17 | 2,4 | 6,3 | 8,7 | 0,7 | 45 | 35 | 38 | 3,1 |
| 1 | 3,3 | 3,7 | 7 | 0,9 | 25 | 80 | 74 | 2,6 |
| 2 | 2,9 | 3,8 | 6,7 | 0,7 | 30 | 105 | 148 | 5,9 |
| 3 | 3,4 | 3,5 | 6,9 | 1 | 27 | 40 | 36 | 2,9 |
| 4 | 2,9 | 4,1 | 7 | 1 | 50 | 32 | 33 | 1,2 |
| 5 | 2,7 | 3,9 | 6,6 | 1,1 | 21 | 50 | 85 | 1,9 |

**Table 4: Serum biochemistries of CanL and healthy dogs**

CanL: Canine leishmaniasis
