# Supplementary material for: MicroRNA-21 and microRNA-148a affects PTEN, NO and ROS in canine leishmaniasis
Source: Front Genet. 2023 Apr 13;14:1106496. doi: 10.3389/fgene.2023.1106496 (PMC10137164; doi:10.3389/fgene.2023.1106496)
Supplement: Supplementary file 8 [file Table6.DOCX]

**Table 6. Comparison between PMA and ROS production in the CanL group**

| Animal | Medium | PMA |
| --- | --- | --- |
| 1 | 352846,90 | 156310,70 |
| 2 | 241700,80 | 503176,30 |
| 3 | 292561,30 | 545522,10 |
| 4 | 142972,40 | 252162,70 |
| 5 | 353418,30 | 468742,60 |
| 6 | 73461,24 | 117498,50 |
| 7 | 48932,90 | 50086,47 |
| 8 | 210101,00 | 571714,30 |
| 9 | 73890,44 | 191610,10 |
| 10 | 232919,80 | 121382,80 |
| 11 | 139981,30 | 393442,60 |
| 12 | 124148,80 | 258129,50 |
| 13 | 198228,90 | 263002,40 |
| 14 | 303754,70 | 318072,90 |
| 15 | 200801,90 | 448691,90 |
| 16 | 135906,30 | 444075,30 |
| 17 | 112695,80 | 257676,70 |
